# Effects of esketamine on postoperative fatigue syndrome in patients after laparoscopic resection of gastric carcinoma: a randomized controlled trial

**DOI:** 10.1186/s12871-024-02513-w

**Published:** 2024-05-24

**Authors:** Xinru Lin, Xiaoxue Feng, Linxiao Sun, Yijian Wang, Xudong Wu, Shufang Lu, Lulu Shao, Wenchao Wang, Liqun Yang, Wujun Geng, Hai Lin

**Affiliations:** 1 Department of Pain, The First Affiliated Hospital of Wenzhou Medical University, Wenzhou, 325000 China; 2 Wenzhou Key Laboratory of Perioperative Medicine (2021HZSY0069), Wenzhou, 325000 China; 3 Key Laboratory of Diagnosis and Treatment of Severe Hepato-Pancreatic Diseases of Zhejiang Province, The First Affiliated Hospital of Wenzhou Medical University, Wenzhou, Zhejiang Province 325000 China; 4 Department of Anesthesiology Renji Hospital, Shanghai Jiaotong University School of Medicine, No.160 Pujian road, Shanghai, 200127 China

**Keywords:** Esketamine, Postoperative fatigue syndrome, Gastric carcinoma, Randomized controlled trial

## Abstract

**Background:**

Despite the implementation of various postoperative management strategies, the prevalence of postoperative fatigue syndrome (POFS) remains considerable among individuals undergoing laparoscopic radical gastrectomy. While the N-methyl-D-aspartic acid receptor antagonist esketamine has demonstrated efficacy in enhancing sleep quality and alleviating postoperative pain, its impact on POFS remains uncertain. Consequently, the objective of this study is to ascertain whether perioperative administration of esketamine can effectively mitigate the occurrence of POFS in patients undergoing laparoscopic radical gastrectomy.

**Methods:**

A total of 133 patients diagnosed with gastric cancer were randomly assigned to two groups, namely the control group (Group C) (*n* = 66) and the esketamine group (Group E) (*n* = 67), using a double-blind method. The Group C received standardized anesthesia, while the Group E received esketamine in addition to the standardized anesthesia. The primary outcome measure assessed was the Christensen fatigue score at 3 days after the surgical procedure, while the secondary outcomes included the disparities in postoperative fatigue, postoperative pain, sleep quality, and adverse reactions between the two groups.

**Results:**

In the group receiving esketamine, the fatigue scores of Christensen on the third day after surgery were significantly lower compared to the Group C (estimated difference, -0.70; 95% CI, -1.37 to -0.03; *P* = 0.040). Additionally, there was a significant decrease in the occurrence of fatigue in the Group E compared to the Group C on the first and third days following surgery (*P* < 0.05). Also, compared to individuals who had distal gastrectomy, those who had entire gastrectomy demonstrated a higher degree of postoperative tiredness reduction with esketamine. Furthermore, the Group E exhibited reduced postoperative pain and improved sleep in comparison to the Group C. Both groups experienced similar rates of adverse events.

**Conclusions:**

The use of esketamine during the perioperative period can improve POFS after laparoscopic radical gastrectomy, without adverse reactions.

**Trial registration:**

Registered in the Chinese Clinical Trial Registry (ChiCTR2300072167) on 05/06 /2023.

## Introduction

Gastric carcinoma ranks as the fifth most prevalent malignant tumor globally, with elevated morbidity and mortality rates. Annually, gastric cancer claims the lives of 700,000 individuals [1, 2]. Presently, laparoscopic radical gastrectomy stands as the principal therapeutic approach for gastric cancer due to its microtraumatic nature and expedited postoperative recuperation [3, 4]. Nevertheless, approximately 90% of patients subjected to laparoscopic radical gastrectomy continue to experience postoperative fatigue syndrome(POFS), a condition marked by fatigue, sleep disruptions, inattentiveness, and other enduring symptoms [5], which can last for days or even months. The delayed recovery process, prolonged hospital stays [6], and diminished quality of life [7] resulting from this hindrance have significantly impeded the successful implementation of enhanced recovery after surgery (ERAS). In order to maximize the overall outcome for these individuals, it becomes imperative to mitigate the occurrence of POFS.

However, due to the intricate nature of POFS, no singular intervention has been identified as efficacious in ameliorating the condition thus far [8]. According to Chen et al. (2015), their research on animals suggests a potential association between postoperative fatigue syndrome (POFS) and excitatory amino acid neurotransmitter receptors, specifically N-methyl-D-aspartic acid receptors (NMDA). They also propose that the use of NMDA receptor antagonists may alleviate central fatigue in POFS patients [9]. However, there is currently no evidence supporting the effectiveness of esketamine, an NMDA receptor antagonist, in improving POFS after laparoscopic radical gastrectomy.

In the context of laparoscopic radical gastrectomy, it is commonly observed that a significant number of patients encounter postoperative pain due to surgical incisions and tissue damage. This pain, along with other traumatic stimuli, prompts peripheral tissues to generate and release diverse inflammatory factors, thereby inducing a state of stress within the body. Consequently, the normal functioning of various bodily systems is affected, leading to a compromised immune system and heightened occurrence of complications [10, 11]. It is plausible to mitigate postoperative fatigue by implementing optimal analgesic measures [6]. Esketamine, the S-enantiomer of ketamine, exhibits greater sedative and analgesic properties compared to ketamine, while also presenting fewer adverse effects [12]. When used as a supplementary agent during general anesthesia, intravenous administration of esketamine has been shown to enhance analgesia, decrease postoperative pain intensity, and reduce the need for opioids [13].

Various factors, such as the surgical procedure and discomfort caused by postoperative drainage tubes, can negatively impact the quality of a patient’s sleep following surgery. Adequate sleep, however, plays a crucial role in expediting post-operative recovery and alleviating fatigue among patients [14]. The sedative and anxiolytic properties of Esketamine have been found to facilitate improved sleep patterns, allowing patients to fall asleep and remain asleep more effortlessly [15]. Additionally, Esketamine has been observed to augment cerebral blood flow, promote the elimination of brain metabolites, and expedite cognitive function recovery, potentially contributing to enhanced sleep quality [16].

Furthermore, esketamine has demonstrated remarkable promise not only in clinical anesthesiology applications but also in the treatment of depression. In fact, the FDA recently approved esketamine in the US for the treatment of patients with depression who have not responded to two or more antidepressant drug trials [17]. In a similar vein, esketamine has a positive impact on psychological distress caused by anxiety and depression in palliative care [18].

Several studies have reported that the administration of Esketamine during the perioperative phase can enhance patients’ overall recovery and facilitate postoperative recuperation [19–21]. Nevertheless, previous research has yielded inconclusive findings regarding the aforementioned effect [22]. Consequently, in order to ascertain the potential benefits of administering esketamine during and after surgery, we undertook a series of prospective, randomized controlled trials. Our hypothesis posited that the utilization of perioperative esketamine would mitigate postoperative pain, enhance sleep quality, and ultimately alleviate the occurrence of postoperative fatigue syndrome.

## Methods

### Study design and participants

The prospective, randomized, double-blind, controlled trial was conducted at the First Affiliated Hospital of Wenzhou Medical University. The study enrolled 133 patients with gastric cancer who were treated by laparoscopic surgery between January 2023 and April 2023 (the diagnostic criteria for gastric cancer are defined in the “Health Industry Standards of the People’s Republic of China: Diagnostic Criteria for Gastric Cancer (WS 316–2010)”), with no gender restriction, age ≥ 18 years old, and ASA grade I to II. The following exclusion criteria were applied: (1) History of radiotherapy and chemotherapy for gastric cancer; (2) Mental disorders; (3) Severe hypertension, coronary heart disease, cardiac insufficiency, pulmonary hypertension, cranial or ocular hypertension, hyperthyroidism, etc.; (4) patients with severe liver and kidney dysfunction; (5) patients with a history of allergic reactions to the drugs used in this study; (6) It is expected that the operation is expected to take more than 10 h, there is the possibility of large blood loss, or conversion to open laparotomy.

This study was approved by the Clinical Research Ethics Committee of the First Affiliated Hospital of Wenzhou Medical University (approval number: KY2022-201). Additionally, all patients provided written informed consent. The trial was registered at the Chinese Clinical Trial Registry, for the record Numbers for ChiCTR2300072167.

### Randomization and blinding

Our research statistician generated random numbers using a computer (simple randomization). The day before the procedure, a study nurse sealed the random numbers in sequentially numbered envelopes and transmitted them to the anesthesiologist. The random numbers then determined the patient’s study group.

Either the esketamine group (Group E) or the control group (Group C) was randomly assigned. Preoperative interviews, eligibility assessments, obtaining written informed consent, the inclusion of participants, and postoperative follow-up were conducted by investigators who had not been involved in perioperative patient care prior to the study and had received training in the assessment of the Visitation scale (all trained and certified by Xinru Lin). Patients and investigators were blinded to study group assignments.

### Intervention

Prior to surgery, all patients were routinely prohibited from drinking and fasting, and no preoperative medications were administered. Following the entry into the operating room, a central vein catheterization was performed(internal jugular vein, Catheter: Bioptimal, cv-501-20yt, size: 5Fr (1.6 mm), Single cavity,20 cm,16Ga), followed by a radial artery puncture catheterization, in order to monitor invasive blood pressure. Patients in both groups receive inhalational and intravenous anesthesia. Under controlled breathing, the Group C was given sufentanil (0.3–0.6 ug/kg), propofol (2 mg/kg), and cisatracurium besylate (0.2 mg/kg) in sequential order during the induction period of anesthesia. The Group E was additionally given 0.5 mg/kg esketamine. During the operation, (0.8-1.0 MAC) sevoflurane was inhaled. Additionally, sufentanil 0.15–0.7ug/kg was intravenously administered at intervals of 30 min. The use of muscle relaxants and vasoactive medications was as required at the discretion of the attending anesthesiologist. The rate of sevoflurane was adjusted intraoperatively to maintain a BIS value of 40–60. Discontinue cisatracurium besylate 30 min before the end of surgery, at the end of the suture, Anesthesia drugs administered inhaled were stopped, and 3 ml of 0.75% ropivacaine was injected into skin wounds to provide anesthesia locally.

After surgery, patients received patient-controlled intravenous analgesia (PCIA). Analgesic pumps in the Group E received sufentanil 2 µg/kg combined with esketamine 1 mg/kg. Group Cs received sufentanil 2 µg/kg, and the total volume of both groups was 100 ml. It was administered continuously to all patients (at a basal rate of 2 mL/h) and on demand as a 2 mL bolus with a lockout interval of 10 min. Immediately after suturing the skin, the infusion began and lasted 48 h. If the VAS score is greater than 3, press the control button, and if the pain does not subside after 30 min, tramadol sustained-release tablets 50–100 mg are used as a remedial analgesic.

### Outcome measures

The main objective of this study was to assess the variation in postoperative fatigue syndrome (POFS) as measured by the Christensen score three days after surgery. The Christensen fatigue scale, widely employed in clinical settings [23], was utilized to evaluate fatigue levels, with a score of ≥ 6 indicating the presence of postoperative fatigue [24]. The Christensen fatigue scale was administered at four time points: one day before surgery, as well as one, three, and seven days after the surgical procedure.

Secondary outcome measures encompass the evaluation of fatigue levels using the 10-item short form of the Identity-Consequence Fatigue Scale (ICFS-10), assessment of pain severity through the VAS pain score, utilization of postoperative relief analgesics, analysis of sleep quality, determination of the first time out of bed, calculation of the duration of hospitalization, and monitoring of adverse events (such as nausea and vomiting, dizziness, intestinal obstruction, hyperthermia, hypertension, delirium, and palpitations) during the PCIA Rate.

The ICFS, a multidimensional measurement tool, is employed in evaluating the fatigue levels and resumption of regular activities among surgical patients [25, 26]. The ICFS-10, a modified version of the original 31-item ICSF scale, effectively captures 98% of the overall fatigue changes from preoperative to postoperative states, thereby serving as a reliable fatigue indicator subsequent to surgery. Additionally, the survey comprises a concise set of 10 questions, facilitating patient completion and enhancing compliance [27]. In the event of hospital discharge within 7 days post-surgery, patients will be contacted either via phone or WeChat.

### Sample size

No similar research has been conducted on patients who have undergone laparoscopic radical gastrectomy, making it challenging to establish an appropriate foundation for determining the sample size. Based on preliminary findings, Christensen scores on day 3 in the Group C was 5.61 ± 1.63(*n* = 6), and that in the Group E was 4.79 ± 1.89 (*n* = 6). Determine the detection level α as 0.05, the detection level β as 0.20, and the degree of certainty (1-β) as 0.80. According to the PASS 15 software, the sample size for the Group E (N1) should be 48 cases, and the sample size for the Group C (N2) should be 48 cases. Considering the 20% loss of follow-up rate, 116 patients, with 58 in each group, were required for this study.

### Statistical analysis

Statistical analysis was performed using SPSS20.0 (IBM, Armonk, NY, USA). Data with a normal distribution were expressed as means ± standard deviations, but data with a skew distribution were expressed as median (25th,75th percentiles). The categorical variable was represented by the number of patients (%). Continuous variables such as Analgesic Dosags were tested for normality using the Kolmogorov-Smirnov test, then Mann-Whitney U or independent sample t tests were used to compare them. Repeated measurement data such as Christensen Score and ICFS-10 score were compared using Repeated ANOVA. The chi-square test or Fisher’s exact test is used to compare categorical variables such as preoperative ASA grading, TNM staging, postoperative fatigue incidence, adverse events, etc., and the results are presented as a percentage. The Hodges-Lehman approach was used to evaluate the differences between the medians and 95% CI. Statistics were deemed significant at *P*<0.05.

## Results

### Participants characteristics

**Fig. 1:**
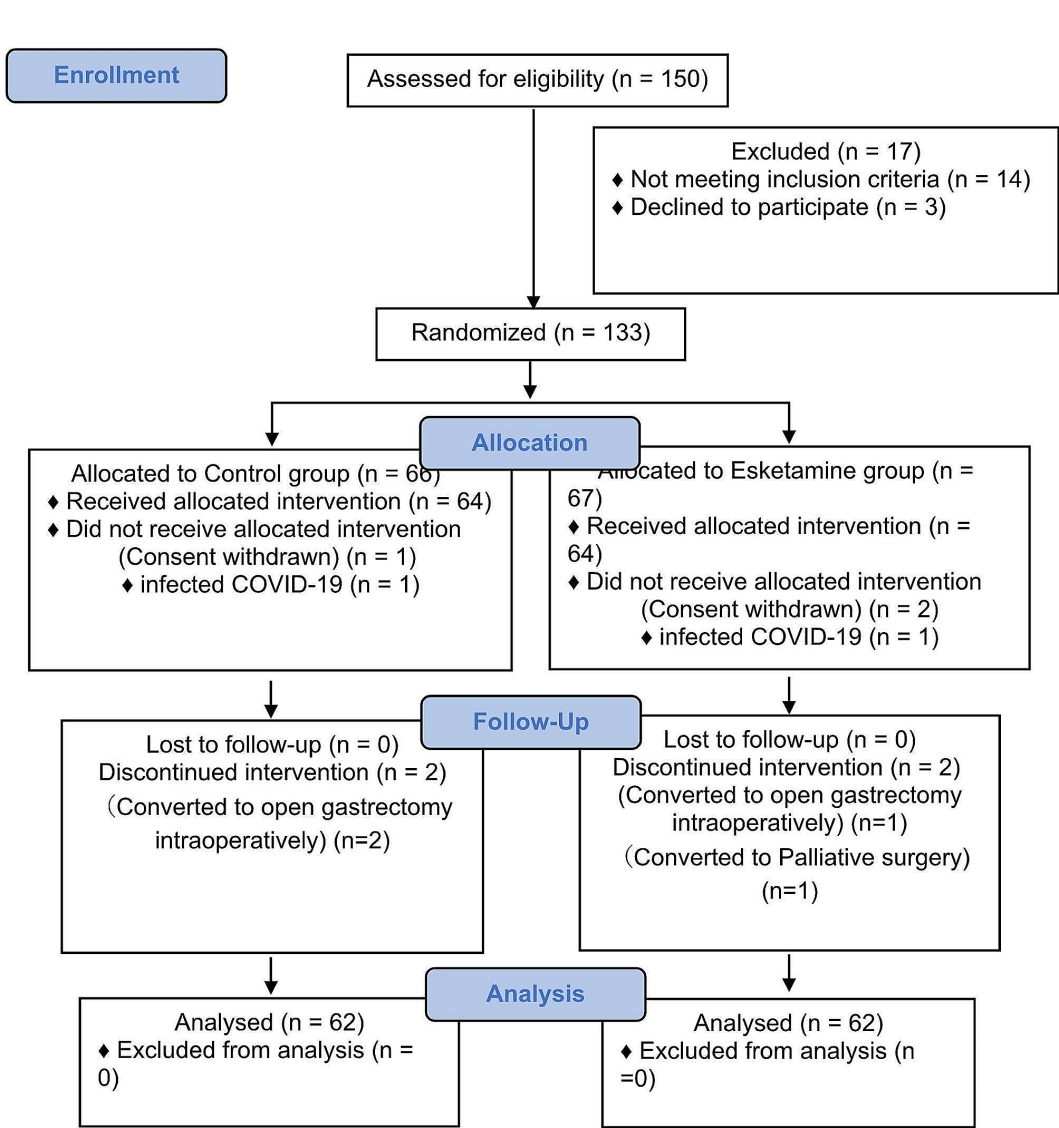
Consolidated Standards of reporting trials flowchart of participant flow

A total of 150 patients were screened, 136 of whom met the inclusion criteria. Three of these individuals refused to participate in the study, in the end, 133 patients were enrolled and randomly assigned to two groups (66 in the Group C and 67 in the Group E). Three patients withdrew consent on the day of surgery: one from the Group C and two from the Group E. There were three patients excluded from the study due to conversion to open surgery (2 in the Group C and 1 in the Group E). Moreover, one patient in the Group E was excluded for palliative surgery. At the same time, two were excluded for delayed operations due to infection with COVID-19(1 in the Group C and 1 in the Group E). In total, 124 patients were enrolled in the primary endpoint analysis (62 in the Group E and 62 in the Group C) (Fig. 1). The two groups were similar in terms of demographic characteristics and perioperative data (Table 1).

**Table 1:** Basic Characteristics and Intraoperative variables

	Group E(*n* = 62)	Control group(*n* = 62)	P value
Age(years)	64.30 ± 10.40	66.81 ± 11.27	0.212
Sex			0.692
Male	45(72.58)	43(72.58)	
Female	17(27.42)	19(27.42)	
BMI (kg/m^2^)	22.56 ± 3.04	22.65 ± 4.00	0.897
SBP (mmHg)	128.05 ± 18.96	127.15 ± 16.58	0.789
Preoperative comorbidities			
Hypertension	31(50.00)	24(38.71)	0.206
Diabetes mellitus	7(11.29)	5(8.06)	0.544
Arrhythmia	23(37.10)	23(37.10)	> 0.999
ASA grade			0.143
I	7(11.29)	13(20.97)	
II	55(88.71)	49(79.03)	
TNM stage			0.440
0	4(6.45)	6(9.68)	
II	25(40.32)	20(32.26)	
II	15(24.19)	11(17.74)	
III	18(29.03)	25(40.32)	
Type of surgery			0.594
Distal gastrectomy	43(69.35)	40(64.52)	
Total gastrectomy	16(25.81)	18(29.03)	
Proximal gastrectomy	3(4.84)	4(6.45)	
Operation time (min)	212.85 ± 52.88	205.60 ± 63.37	0.490
Anaesthesia time (min)	259.00(216.00,295.00)	242.50(200.00,292.00)	0.355
Intra-operative			
sufentanil used (ug/kg)^*a*^	35.05 ± 15.11	32.58 ± 11.25	0.304
infusion(ml)	24.36(18.04,29.61)	24.17(18.25,31.55)	0.914
Hypertension^*a*^	4(6.45)	1(1.61)	0.361
Hypotension^*b*^	25(40.32)	26(41.94)	0.855
Estimated blood loss(ml)	38.61 ± 11.41	36.53 ± 14.10	0.368
Blood transfusion cases	1(1.61)	1(1.61)	> 0.999
Wound infection	2(3.23)	1(1.61)	> 0.999
Intestinal hemorrhage	0(0)	1(1.61)	> 0.999
Intestinal perforation	0(0)	0(0)	/

The data are presented as mean ± SD, medians (25th, 75th), or the number of patients and percentage (%)

The P values were calculated by the t test, Mann–Whitney U test, chi-square test, or Fisher exact test

*a* Systolic an increase in systolic blood pressure of more than 30% from the baseline (the ward’s average value) or blood pressure greater than 180 mmHg that required intravenous agents to decrease blood pressure, such as metoprolol, urapidil, or nitroglycerin

*b* Systolic a decrease in systolic blood pressure of more than 30% from the baseline (the ward’s average value) or blood pressure less than 90 mmHg that required intravenous vasopressors, such as dopamine, norepinephrine, or ephedrine

### Primary outcome

**Fig. 2:**
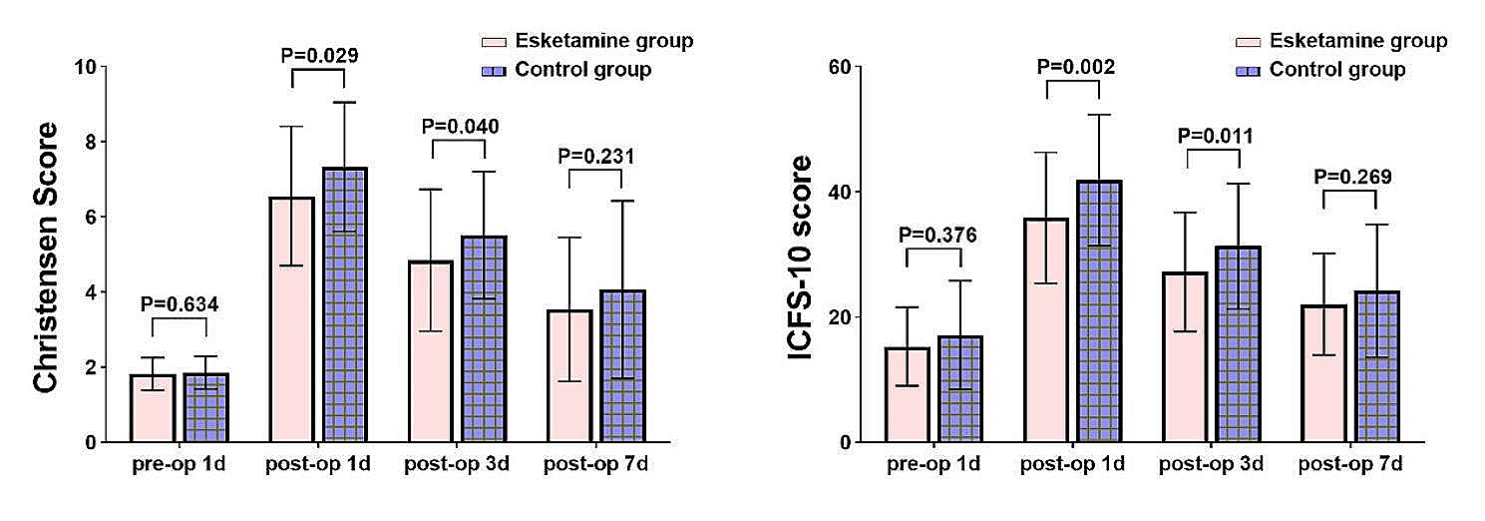
Histogram of differences in christensen fatigue scores and ICFS-10 scores between two groups

All patients’ postoperative Christensen scores increased from their initial values. In comparison to the Group C, the Group E scored lower on the Christensen scale on days 1 and 3 (estimated difference, -0.76; 95% CI, -1.44 to 0.08; *P* = 0.029) and (estimated difference, -0.70; 95% CI, -1.37 to -0.03; *P* = 0.040) (Table 2). Additionally, the ICFS-10 scores for the Group E were higher than those for the Group C on days 1 and 3 following surgery (estimated difference, -6.43; 95% CI, -10.41 to -2.45; *P* = 0.002) and (estimated difference, -5.05; 95% CI, -8.90 to -1.19; *P* = 0.011) (Fig. 2). At 1 day after surgery, the Group E’s fatigue incidence rate was 37 (59.68%), the Group C’s was 53 (85.48%), and at 3 days after surgery, it was 21 (33.87%), the Group C’s was 33 (53.23%), both with a P value < 0.05 (Table 2)( Fig. 2).

Considering the potential impact of the type of surgery (distal gastrectomy versus total gastrectomy) on POFS, a sub-analysis comparing distal gastrectomy vs. total gastrectomy in terms of Christensen score was performed. On the initial postoperative day, patients in group E who had either a total gastrectomy or a distal gastrectomy had lower Christensen scores than patients in group C (estimated difference, -1.19; 95% CI, -2.12 to -0.26; *P* = 0.014) and (estimated difference, -0.96; 95% CI, -1.80 to -0.12; *P* = 0.026). On the third day after surgery, compared to group C, the Christensen score of patients undergoing total gastrectomy in group E was lower (estimated difference, -1.38; 95% CI, -2.48 to 0.28; *P* = 0.016). Patients receiving distal gastrectomy in group E showed a reduced Christensen score on the third postoperative day compared to group C, but no statistically significant difference was revealed (Table 2).

The Christensen score, and ICFS-10 score in the ketamine group were significantly lower than those in the control group on day 1 and day 3 after surgery.

**Table 2:** Comparison of postoperative fatigue (POF)

	Group E(*n* = 62)	Group C(*n* = 62)	Difference(95% CI)	P value
Christensen Score				
Preoperative 1d	1.81 ± 0.45	1.85 ± 0.44	-0.04(-0.21,0.13)	0.634
Postoperative 1d	6.66 ± 1.85	7.42 ± 1.68	-0.76(-1.44,-0.08)	0.029
Postoperative 3d	4.91 ± 1.86	5.62 ± 1.63	-0.70(-1.37,-0.03)	0.040
Postoperative 7d	3.55 ± 1.94	4.07 ± 2.36	-0.51(-1.36,0.33)	0.231
Perioperative period	4.23 ± 1.23	4.74 ± 1.24	-0.50(-0.97,-0.04)	0.034^*a*^
Distal Gastrectomy^*b*^				
Preoperative 1d	1.84 ± 0.44	1.90 ± 0.44	-0.06(-0.26,0.14)	0.539
Postoperative 1d	6.12 ± 1.95	7.08 ± 1.67	-0.96(-1.80,-0.12)	0.026
Postoperative 3d	4.66 ± 1.89	5.23 ± 1.66	-0.57(-1.39,0.25)	0.170
Postoperative 7d	3.32 ± 1.83	3.59 ± 2.23	-0.27(-1.26,0.73)	0.593
Total Gastrectomy^*c*^				
Preoperative 1d	1.75 ± 0.45	1.72 ± 0.46	0.03(-0.29,0.35)	0.860
Postoperative 1d	7.20 ± 1.37	8.39 ± 1.24	-1.19(-2.12,-0.26)	0.014
Postoperative 3d	5.07 ± 1.75	6.44 ± 1.34	-1.38(-2.48,-0.28)	0.016
Postoperative 7d	3.79 ± 1.89	5.28 ± 2.27	-1.49(-3.03,0.05)	0.057
ICFS-10 score^*d*^				
Preoperative 1d	15.67 ± 6.46	17.04 ± 8.57	-1.36(-4.40,1.68)	0.376
Postoperative 1d	36.50 ± 10.06	42.93 ± 10.08	-6.43(-10.41,-2.45)	0.002
Postoperative 3d	26.85 ± 9.13	31.89 ± 10.26	-5.05(-8.90,-1.19)	0.011
Postoperative 7d	21.96 ± 8.09	24.07 ± 10.63	-2.12(-5.89,1.66)	0.269
Perioperative period	25.25 ± 6.58	28.98 ± 6.59	-3.74(-6.33,-1.15)	0.005^*a*^
POF^*e*^				
Postoperative 1d	37(59.68)	53(85.48)	0.25(0.11,0.60)^*f*^	0.001
Postoperative 3d	21(33.87)	33(53.23)	0.45(0.22,0.93)^*f*^	0.030
Postoperative 7d	17(27.42)	21(33.87)	0.74(0.34,1.59)^*f*^	0.436

The data are presented as mean ± SD or the number of patients and percentage (%)

The P values were calculated by the Multiple measurement analysis of variance or chi-square test

*a* Represents the P value for total comparison between Group E and Group C

*b* Represents the comparison of Christensen score between two groups of distal gastrectomy patients

*c* Represents the comparison of Christensen score between two groups of total gastrectomy patients

*d* Represents the 10-item short form of the Identity-Consequence Fatigue Scale

*e* Christensen score ≥ 6 indicates postoperative fatigue (POF), the data are presented as the number of patients and percentage (%)

*f* Represents Odds Ratio (95% CI)

### Secondary outcome

The VAS pain levels of the Group E were substantially lower at 1 and 3 days following surgery as compared to the Group C (estimated difference, -1.09; 95% CI, -2.01 to -0.16; *P* = 0.022) and (estimated difference, -0.79; 95% CI, -1.33 to -0.25; *P* = 0.005) (Table 3). Additionally, within 48 h after surgery, the Group E used considerably less remedial analgesic (estimated difference, -10.86; 95% CI, -20.97 to -0.74; *P* = 0.036) (Table 3). The Sleep score of the Richards-Campbell Sleep Questionnaire in the Group E was significantly lower than that in the Group C on day 1, 3 and 7 after surgery (estimated difference, -5.79; 95% CI, -10.55 to -1.02; *P* = 0.018), (estimated difference, -5.01; 95% CI, -9.38 to -064; *P* = 0.025) and (estimated difference, -4.19; 95% CI, -7.68 to -0.70; *P* = 0.019) (Table 3). There was also a reduction in the first time to get out of bed in the Group E compared to the Group C (*P* < 0.05) (Table 3).

Between the Group E and the Group C, there was no discernible difference in the incidence of postoperative adverse reactions such as nausea, vomiting, dizziness, intestinal obstruction, hypertension, increased body temperature, delirium, and palpitations (all *P* > 0.05) (Table 4).

**Table 3:** Postoperative Variables

	Group E(*n* = 62)	Group C(*n* = 62)	Difference(95% CI)	P value
VAS^*a*^				
Postoperative 1d	6.55 ± 2.66	7.64 ± 2.16	-1.09(-2.01,-0.16)	0.022
Postoperative 3d	2.69 ± 1.50	3.48 ± 1.31	-0.79(-1.33,-0.25)	0.005
Postoperative 7d	1.96 ± 1.37	1.86 ± 1.29	0.10(-0.41,0.61)	0.707
RCSQ^*b*^				
Preoperative 1d	17.65 ± 11.57	19.43 ± 12.26	-1.78(-6.23,2.67)	0.431
Postoperative 1d	25.84 ± 12.02	31.62 ± 13.70	-5.79(-10.55,-1.02)	0.018
Postoperative 3d	19.21 ± 10.12	24.22 ± 13.07	-5.01(-9.38,-0.64)	0.025
Postoperative 7d	15.63 ± 7.30	19.82 ± 10.32	-4.19(-7.68,-0.70)	0.019
Postoperative period			-0.59(-1.06,-0.13)	0.013^*c*^
Analgesic Dosage(mg/kg)^*d*^	33.41 ± 25.03	44.27 ± 24.51	-10.86(-20.97,-0.74)	0.036
First time out of bed (days)	2.76 ± 0.81	3.43 ± 1.22	-0.67(-1.01,-0.27)	0.001
Length of stay (days)	10.04 ± 3.44	11.72 ± 4.83	-1.68(-3.28,-0.08)	0.040

The data are presented as mean ± SD

The P values were calculated by the t test, t’ test

*a* Represents Visual Analogue Scale/Score

*b* represent Richards-Campbell Sleep Questionnaire

*c* Represents the P value for total comparison between Group E and Group C

*d* Expressed as morphine equivalent consumption within 48 h after surgery

**Table 4:** Comparison of adverse events between group E and group C

	Group E(*n* = 62)	Group C(*n* = 62)	Difference (95% CI)	P value
Intraoperative events				
Hypotension^*a*^	15(24.19)	16(25.81)	0.92(0.41–2.07)	0.836
Hypertension^*b*^	4(6.45)	1(1.61)	4.2(0.46–38.76)	0.361
Allergic reaction	0(0)	0(0)	/	/
Postoperative events^*c*^				
Nausea and Vomiting	3(4.84)	7(11.29)	0.40(0.10–1.62)	0.187
Dizziness	2(3.23)	2(3.23)	1.00(0.14–7.33)	> 0.999
Intestinal Obstruction	0(0)	1(1.61)	0.98(0.95–1.01)	> 0.999
Hyperthermia	2(3.23)	3(4.84)	0.66(0.11–4.07)	> 0.999
Hypertension	9(14.52)	8(12.90)	1.15(0.41–3.20)	0.794
Delirium	1(1.61)	0(0)	1.02(0.99–1.05)	> 0.999
Palpitation	2(3.23)	1(1.61)	2.03(0.18–23.02)	> 0.999

The data are presented as the number of patients and percentage (%)

The P values were calculated by chi-square test, or Fisher exact test

*a* Systolic a decrease in systolic blood pressure of more than 30% from the baseline (the ward’s average value) or blood pressure less than 90 mmHg that required intravenous vasopressors, such as dopamine, norepinephrine, or ephedrine

*b* Systolic an increase in systolic blood pressure of more than 30% from the baseline (the ward’s average value) or blood pressure greater than 180 mmHg that required intravenous agents to decrease blood pressure, such as metoprolol, urapidil, or nitroglycerin

*c* Postoperative complications are those that occur during the entire hospitalization period following the recovery from anesthesia

## Discussion

Despite advancements in anesthetic management and surgical technique, patients undergoing laparoscopic radical gastrectomy persistently experience postoperative fatigue syndrome. In recent years, clinicians have employed various strategies to mitigate postoperative fatigue, aiming to enhance surgical rehabilitation, minimize hospitalization duration, and alleviate the aforementioned syndrome. However, these endeavors have not yielded any discernible therapeutic advantages. In this clinical trial, the efficacy of esketamine, an NMDA receptor antagonist, in reducing fatigue was evaluated.

Previous studies have established that medications acting as NMDA receptor antagonists can contribute to fatigue reduction in this patient population through various mechanisms, including pain alleviation, tissue damage minimization, attenuation of inflammatory responses [28], improvement of sleep quality, and inhibition of sensitization processes in the nociceptive pathway [29]. Since esketamine’s analgesic effects are one of the ways it improves fatigue, we gave both patient groups the same dosage of sufentanil to guarantee that they maintained the same baseline throughout the experiment. This allowed us to assess the effect of esketamine more precisely. The findings of this trial indicate that in comparison to the Group C, patients in the Group E exhibited reduced levels of fatigue on the initial day following surgery, and a notable decline in fatigue on the third day post-surgery. Additionally, patients in the Group E reported diminished postoperative discomfort, improved sleep quality, and a shorter duration until first ambulation. Adverse events were infrequent and did not display significant differences between the two study groups. These findings suggest that the administration of esketamine during surgical procedures yields considerable advantages in mitigating postoperative fatigue syndrome.

Esketamine, an NMDA receptor antagonist similar to ketamine, exhibits binding affinity to opiate receptors and modulates serotonin and norepinephrine levels in the brain, thereby exerting analgesic, anti-inflammatory, and neuroprotective effects [30]. Extensive investigation has been conducted to elucidate the role of esketamine in laparoscopic surgery. A randomized, double-blind, multicenter study involving 278 patients demonstrated that the combined administration of esketamine and propofol anesthesia synergistically reduced postoperative nausea, vomiting, and other short-term adverse events, thereby enhancing the safety and satisfaction of Enhanced Recovery After Surgery (ERAS) in laparoscopic patients [31]. Similarly, in a related randomized controlled research study, it was observed that patients undergoing laparoscopic surgery who received intraoperative esketamine infusion exhibited improved postoperative sleep quality [21]. A meta-analysis of 12 randomized trials further revealed that the utilization of intravenous esketamine as an adjunct to general anesthesia resulted in reduced pain severity and decreased opioid requirements immediately following surgery [13]. Consequently, based on these findings, it can be inferred that the implementation of esketamine during the preoperative phase is advantageous for the postoperative recovery of patients undergoing laparoscopic surgery.

Studies conducted on POFS rats with resected small intestine have provided evidence that NMDA receptor antagonists have the potential to alleviate fatigue [32]. The efficacy of ketamine in reducing fatigue has been explored in a single clinical study. As reported by Zhao et al. (2022), a solitary subanesthetic dose of ketamine effectively decreased POFS without inducing any adverse postoperative effects [33]. In line with these findings, our investigation revealed that patients who received perioperative esketamine exhibited significantly lower levels of Christensen fatigue on the third day post-surgery compared to the Group C (estimated difference, -0.70; 95% CI, -1.37 to -0.03; *P* = 0.040). Furthermore, it was found that the ICFS-10 score of the Group E exhibited a notable improvement compared to the Group C on the third day post-surgery. (estimated difference, -5.05; 95% CI, -8.90 to -1.19; *P* = 0.011). Furthermore, compared to patients undergoing distal gastrectomy, individuals undergoing whole gastrectomy showed a greater reduction in postoperative fatigue.

The primary objectives of managing postoperative fatigue syndrome involve minimizing hospital stays and expediting patient recovery. Notably, a significant decrease in the duration of hospitalization was observed in the Group E (estimated difference, -1.68; 95% CI, -3.28 to -0.08; *P* = 0.040). Additionally, patients in the Group E reported lower VAS pain scores following surgery, particularly on the third day (estimated difference, -0.79; 95% CI, -1.33 to -0.25; *P* = 0.005). The Group E demonstrated a statistically significant reduction in postoperative analgesic medication compared to the Group C (*P*<0.05). In the gastrointestinal surgery unit, the utilization of sufentanil necessitates an anesthetic prescription from the anesthesiology department. In contrast, tramadol sustained-release tablets are consistently accessible, ensuring prompt administration to alleviate postoperative discomfort among patients. Furthermore, our preliminary experimental findings indicate that tramadol effectively mitigates acute pain following laparoscopic radical gastrectomy. Consequently, tramadol was chosen instead of sufentanil as the postoperative remedial analgesic.

Additionally, the quality of sleep among patients was found to be a significant factor in postoperative recovery within enhanced rehabilitation programs [34]. The survey revealed that the Group E exhibited significantly lower scores on the Richards-Campbell Sleep Questionnaire postoperatively (*P*<0.05). These findings suggest that perioperative administration of esketamine may effectively alleviate pain, enhance sleep quality, and facilitate patient recovery, ultimately leading to shorter hospital stays.

Various adverse effects, such as nausea, vomiting, dizziness, intestinal obstruction, hypertension, hyperthermia, delirium, and palpitations, may occur during the perioperative administration of esketamine [35, 36]. A review and meta-analysis indicate that the incidence of these adverse events is not significantly higher in patients using esketamine compared to those receiving placebos [37, 38]. This aligns with our research findings. Despite exhibiting sympathomimetic effects, esketamine did not elicit any significant difference in intraoperative or postoperative hypertension or hypotension between the two patient groups. This was attributed to the meticulous perioperative management employed for our patients. Moreover, the absence of intergroup disparities and a minimal occurrence of adverse events pertaining to the gastrointestinal system (nausea, vomiting, intestinal obstruction), the central nervous system (delirium), and the circulatory system (palpitation) among the participants of this trial suggests that the utilization of esketamine in perioperative interventions does not seem to augment the likelihood of postoperative complications.

The study is subject to various limitations. Primarily, rather than going with a multi-center design, we went with a monocentric design. The monocentric design contributes to uniformity and standardization in data collection and research methods. In addition, two patients were excluded from the analysis due to contracting COVID-19 subsequent to enrollment, which could have been prevented if randomization had been postponed until the day of the operation. Third, our analysis was limited to esketamine’s impact on POFS. It is theoretically possible for all NMDA receptor antagonists to alleviate central fatigue. For instance, xenon, which inhibits NMDA receptors to provide anesthetic and analgesic effects, has neuroprotective and cardiovascular stabilizing qualities; as a result, it may potentially be helpful for POFS. Since it is difficult and costly to extract xenon, a noble gas, greater investigation into the potential benefits of better NMDA receptor antagonists for POFS treatment is warranted. Furthermore, as this study solely assessed a single dosage of esketamine, the optimal protective dose remains undetermined.

## Conclusion

In conclusion, the utilization of esketamine during the perioperative phase has demonstrated potential in mitigating the occurrence of postoperative fatigue syndrome, alleviating postoperative pain, enhancing sleep quality, facilitating early ambulation, and exhibiting a lack of adverse reactions. Consequently, it is evident that the perioperative administration of esketamine constitutes an efficacious therapeutic approach that should be accessible to individuals undergoing laparoscopic gastric cancer surgery. Moreover, there is an imperative need to ascertain the optimal dosage of esketamine for perioperative antifatigue purposes.

## Data Availability

Upon reasonable request, the corresponding author will provide the data that back up the study’s conclusions.
